# Planctomycetes as Novel Source of Bioactive Molecules

**DOI:** 10.3389/fmicb.2016.01241

**Published:** 2016-08-12

**Authors:** Ana P. Graça, Rita Calisto, Olga M. Lage

**Affiliations:** ^1^Departamento de Biologia, Faculdade de Ciências, Universidade do PortoPorto, Portugal; ^2^CIIMAR—Centro Interdisciplinar de Investigação Marinha e Ambiental—Universidade do PortoPorto, Portugal

**Keywords:** planctomycetes, antibiotic activity, antifungal activity, PKS and NRPS genes, screening, secondary metabolite, genome mining

## Abstract

Marine environments are a fruitful source of bioactive compounds some of which are the newest leading drugs in medicinal therapeutics. Of particular importance are organisms like sponges and macroalgae and their associated microbiome. *Planctomycetes*, abundant in macroalgae biofilms, are promising producers of bioactive compounds since they share characteristics, like large genomes and complex life cycles, with the most bioactive bacteria, the *Actinobacteria*. Furthermore, genome mining revealed the presence of secondary metabolite pathway genes or clusters in 13 analyzed *Planctomycetes* genomes. In order to assess the antimicrobial production of a large and diverse collection of *Planctomycetes* isolated from macroalgae from the Portuguese coast, molecular, and bioactivity assays were performed in 40 bacteria from several taxa. Two genes commonly associated with the production of bioactive compounds, nonribosomal peptide synthetases (NRPS), and polyketide synthases (PKS) genes were screened. Molecular analysis revealed that 95% of the planctomycetes potentially have one or both secondary bioactive genes; 85% amplified with PKS-I primers and 55% with NRPS primers. Some of the amplified genes were confirmed to be involved in secondary metabolite pathways. Using bioinformatic tools their biosynthetic pathways were predicted. The secondary metabolite genomic potential of strains LF1, UC8, and FC18 was assessed using *in silico* analysis of their genomes. Aqueous and organic extracts of the *Planctomycetes* were evaluated for their antimicrobial activity against an environmental *Escherichia coli, E. coli* ATCC 25922, *Pseudomonas aeruginosa* ATCC 27853, *Staphylococcus aureus* ATCC 25923, *Bacillus subtilis* ATCC 6633, and a clinical isolate of *Candida albicans*. The screening assays showed a high number of planctomycetes with bioactive extracts revealing antifungal (43%) and antibacterial (54%) activity against *C. albicans* and *B. subtilis*, respectively. Bioactivity was observed in strains from *Rhodopirellula lusitana, R. rubra, R. baltica, Roseimaritima ulvae*, and *Planctomyces brasiliensis*. This study confirms the bioactive capacity of *Planctomycetes* to produce antimicrobial compounds and encourages further studies envisaging molecule isolation and characterization for the possible discovery of new drugs.

## Introduction

Diseases like cancer and antibiotic resistance impose us a pressing need for the discovery of new effective leads in their treatment. Since the discovery of penicillin, the miracle drug of the twentieth century by Fleming in 1928, we assisted to a boom of molecules release with antibiotic capacity. Due to excessive and incorrect use of antibiotics and the capacity of bacteria to escape their action, we witnessed, in the last decade, to a dramatic increase of bacterial pathogens presenting multidrug resistance to antibacterial agents (Roca et al., 2015). The search for novel bioactive molecules has been essentially based on terrestrial organisms. However, in the last decades, attention has been paid to marine samples. The unique characteristics of the marine environment allied to its unexplored and unknown biologic diversity makes the marine habitats a potential great source of new bioactive molecules. Moreover, microorganisms, in their adaptation to a multitude of different and sometimes extreme marine conditions, are holders of a myriad of metabolic pathways including secondary metabolite activity that are not found in terrestrial ecosystems (Karabi et al., 2016). Furthermore, they are easily and sustainably cultivated in large scale at a reasonable cost which are good characteristics for technological exploitation (Waites et al., 2001; Debbab et al., 2010).

Bioactive compounds, often produced as secondary metabolites, can be, for example alkaloids, sugars, steroids, terpenoids, peptides, and polyketides (Simmons et al., 2005). These substances are mainly produced as a defense strategy and can be used by man as antibacterial, antifungal, antiviral, antitumor, and immunosuppressive among other potential medicines (Laport et al., 2009). The production of secondary metabolites involves complex molecular structures and biochemical pathways (Hutchinson, 2003). Nonribosomal peptides and polyketides are biocompounds synthesized by two classes of enzymes: the nonribosomal peptides synthetases (NRPS) and polyketide synthases (PKS), respectively (Grozdanov and Hentschel, 2007). These enzymes are responsible for many secondary metabolites that exhibit an important biological activity and may be valuable drugs (Hutchinson, 2003; Ansari et al., 2004; Kennedy et al., 2007). These pathway systems have been studied and described mainly in *Actinobacteria* but are also present in other bacterial taxa and even in filamentous fungi and plants (Ayuso-Sacido and Genilloud, 2005). PKS and NRPS genes are codified by gene clusters presents in the genome of various groups of bacteria (Donadio et al., 2007; Graça et al., 2013, 2015).

Planctomycetes are a bacterial phylum with very particular features that make them good candidates for the production of novel bioactive molecules. They possess complex life cycles and quite large genomes for prokaryotes, characteristics that are typical of bacteria known for their bioactive potential like *Streptomyces* and *Myxobacteria* (Jeske et al., 2013). In their adaptation to a wide range of habitats, including extreme environments (Lage and Bondoso, 2014), planctomycetes developed diversified metabolisms still unexploited. Other characteristics make them unique. These include a complex cell plan (Lage et al., 2013; Santarella-Mellwig et al., 2013), polar budding of many members (Ward et al., 2006), and the absence of the bacterial division protein FtsZ (Pilhofer et al., 2008), the presence of membrane coat-like proteins (Santarella-Mellwig et al., 2010), and endocytosis (Lonhienne et al., 2010). Although much is still unknown about planctomycetes secondary metabolism, some recent studies have recently pointed out their bioactive potential. Donadio et al. (2007) analyzed the genome of *Rhodopirellula baltica* SH1255 and verified the presence of two small NRPSs, two monomodular PKSs, and a bimodular NRPS-PKS which may be involved in the synthesis of five different, unknown products. In Antarctic sediments, Zhao et al. (2008) identified planctomycetal type I polyketide synthase domains. Using a comprehensive genome mining approach for the analyses of 13 genomes, Jeske et al. (2013) found 102 candidate genes or clusters.

In our study, we explored the bioactive potential of a unique collection of planctomycetes isolated from the biofilm of macroalgae by genome mining, as well as PKS-I and NRPS gene molecular analyses and antimicrobial bioactivity screenings. As biofilms are complex highly dynamic structured ecosystems where strong chemical competition occurs and planctomycetes are relatively slow growing bacteria, these bacteria should possess well-equipped chemical machinery to be able to fight for their survival and impose themselves in such a competing environment.

## Materials and methods

### Biological material

The 40 planctomycetes under study, belonging to 10 taxa, were isolated from macroalgae surfaces biofilms within the scope of several diversity and phylogenetic studies carried out in rocky beaches in north of Portugal (Lage and Bondoso, 2011). The bacteria were maintained in pure culture at 25°C in M600/M14 agar medium (Lage and Bondoso, 2011). The phylogenetic relationship of the strains are shown in a phylogenetic tree constructed using the sequences available in GenBank database. The sequences of UC49.2 and FC9.2 were assigned with the following accession numbers—KX495344–KX495345. The sequences were aligned using clustalW, and a final maximum likelihood (ML) phylogenetic tree was generated using the aligned 1316 bp applying the general time reversible model with gamma distributed with invariant sites (G+I) rates in MEGA6.06.

The panel of target microorganisms used in the screening assays were an environmental strain of *E. coli* (Cabral and Marques, 2006), *E. coli* ATCC 25922, *Pseudomonas aeruginosa* ATCC 27853, *Staphylococcus aureus* ATCC 25923, *Bacillus subtilis* ATCC 6633, and a clinical isolate of *C. albicans*.

### Amplification and sequencing of PKS-I and NRPS genes

Total genomic DNA was extracted from pure cultures using the E.Z.N.A. Bacterial DNA Isolation Kit (Omega), following the recommended instructions. In order to perform the screening of the genomic bioactive potential the degenerate primers MDPQQRf (5′-RTRGAYCCNCAGCAICG-3′) and HGTGTr (5′-VGTNCCNGTGCCRTG-3′) and MTRF2 [5′-GCNGG(C/T)GG(C/T)GCNTA(C/T)GTNCC-3′] and DKF (5′-GTGCCGGTNCCRTGNGYYTC-3′) were used for the amplification of PKS-I (Kim et al., 2005) and NRPS (Neilan et al., 1999), respectively. These primers amplify for the alpha keto syntethase of PKS-I and core motif-V of NRPS genes. The amplification mixture was composed of 12.5 μL of NZYTaq 2 × Green Master Mix, 2 μL of each primer, 1 μL genomic DNA as template, and completed with sterilized ultrapure water to a final volume of 25 μL. The amplification parameters for both primers were identical and the PCR amplifications were performed in a MyCycler™ Thermo Cycler (Bio-Rad) as described in Graça et al. (2013). The PCR products were visualized after electrophoresis in a 1.2% agarose gel with 1 × TAE buffer in a VWR GenoPlex. The expected PCR products size of PKS-I and NRPS genes were 750 and 1000 bp, respectively.

Bands with several sizes were excised and purified using Illustra GFX PCR DNA and Gel Band Purification Kit (GE Healthcare) and directly sequenced using appropriate primers. The sequences were manually corrected and consensus constructed by means of Vector NTI Advance 11.5.3. Data used for UC8 PKS-I was the portion of the annotated genome that matched with the amplicon sequenced, with additional 200 bp above and after alignment, in order to retrieve more information for the analysis.

### *In silico* analysis of PKS-I and NRPS genes

The resulting consensus sequences of the strains were searched through Basic Local Alignment Search Tool (BLAST), classified in the Nucleotide collection of the National Center of Biotechnology Information (NCBI) based on somewhat similarity. Information regarding similarity and coverage values was assessed for the validation of the amplification of the targeted genes.

Natural Product Domain Seeker—NaPDoS, a bioinformatic tool to search for PKS and NRPS modules and to predict bioactive pathway products, was used based on the FASTA file with the nucleotide sequences choosing Predicted coding sequences or PCR products. The sequences were screened to detect and extract C- (NRPS) and KS- (PKS) domains and candidate secondary metabolites domains (Ziemert et al., 2012).

Blast2go is a fast bioinformatics tool that uses several public databases to assign a biological function to a sequence by identifying similar characterized sequences. This tool was used to compare and confirm results obtained by manual search and NaPDoS. Gene synteny of the results obtained in the prediction of the metabolite pathways were analyzed in SynTax (Oberto, 2013). Nucleotide sequences of the genes were translated into amino acidic sequences using ExPASy tool and the longer sequences translated were used to construct a phylogenetic tree. Phylogenetic and molecular evolutionary analyses were conducted using MEGA version 6 (Tamura et al., 2013). Sequences were aligned using clustalW, and a final ML phylogenetic tree was generated applying Equal input model after searching for the fittest model also in MEGA6.06. Sequences with small size were removed from the analysis.

The sequences used in this study were submitted to GenBank database and the accession number assigned for PKS and NRPS gene sequences were KX306801–KX306815. Since some sequences did not reach enough size to be submitted in this database they are provided as Supplementary Material (Annex I).

### Secondary metabolite related *in silico* analysis of planctomycete genomes

The genomes of strains *Rubripirellula obstinata* LF1^T^ (GenBank: LWSK00000000), *Roseimaritima ulvae* UC8^T^ (GenBank: LWSJ00000000) and *Planctomycetes* strain FC18 (GenBank: LWSI00000000) used in this study were previously curated and annotated (unpublished results). The contigs were searched for their genomic bioactive potential using antibiotics and Secondary Metabolites Analysis Shell—antiSMASH 3.0 (Weber et al., 2015) and NaPDoS annotation pipelines. The antiSMASH analysis retrieved information of the biosynthetic clusters and of biosynthetic, transport, and regulatory genes present in the submitted genomes by gathering data of several *in silico* secondary metabolite analyses tools. The NaPDoS tool was used as referred above but using the Genome or metagenome contigs (DNA) option. These two tools were used as a complement and for data confirmation.

### Antimicrobial screenings

The screening of antimicrobial activity was performed in 35 planctomycetes (Table 1). Actively growing strains were initially pre-cultured in M600 medium, at 25°C, 220 r.p.m. for 2 days. Each planctomycete was then incubated in M600 and M607 media (Lage and Bondoso, 2011) for 7 days, at 25°C and 220 r.p.m. Each culture was then used to prepare four types of bacterial extracts: 1 aqueous and 3 organic. The organic extracts, for which a mixture of acetone plus 10% DMSO was used, were obtained from culture broth (A/C), cell pellet (A/P), and supernatant (A/S). Two milliliters of culture were used for the A/C; another 2 mL of culture were centrifuged at 13,300 r.p.m. for 5 min and the supernatant (A/S) and pellet (A/P) collected. To these three samples, 2 ml of the solvent mixture were added. These mixtures were incubated for 1 h under continuous shaking and 2 mL of the upper phase (organic) were removed to a new tube and dried to half volume (twice concentrated). To the A/P extracts, sterilized distilled water was added up to 1 mL. To obtain the aqueous supernatant extract (F), 2 mL of culture were centrifuged at 13,300 r.p.m. for 5 min and the supernatant filter sterilized through a 0.22 μm (Graça et al., 2015).

**Table 1:** **Results of the molecular analysis and screening of the bioactive potential of the studied Planctomycetes**.

**Strain**	**Genera**	**Bioactivity against *Candida albicans***	**Bioactivity against *Bacillus subtilis***	**PKS-I size amplicon**	**NRPS size amplicon**	**PKS-I genes closest similar result using blastn in NCBI**	**Similarity; Coverage**	**PKS-I genes closest similar result using blastp in NCBI**	**Similarity; Coverage**	**NaPDoS prediction pathway product**	**NRPS genes closest similar result using blastn in NCBI**	**Similarity; Coverage**	**NRPS genes closest similar result using blastp in NCBI**	**Similarity; Coverage**	**NaPDoS prediction pathway product**
Rb SH1	*Rhodopirellula baltica*	Bioactive	Bioactive	750 bp	1000 bp	*Rhodopirellula baltica* SH 1 complete genome; segment 22/24	99%; 99%	polyketide synthase [*Rhodopirellula baltica*]	100%; 97%	epothilone	NSR	–	–	–	–
UC21	*Rhodopirellula baltica*	Bioactive	Bioactive	750 bp	–	–	–	–	–	–	–	–	–	–	–
UC49.1	*Rhodopirellula baltica*	Bioactive	Bioactive	750 bp	1000 bp	*Rhodopirellula baltica* SH 1 complete genome; segment 22/24	97%; 100%	polyketide synthase [*Rhodopirellula baltica*]	100%; 97%	epothilone	–	–	–	–	–
FC9.2	*Rhodopirellula* sp.	Bioactive	Bioactive	–	750 bp	Alpha proteobacterium F16 beta ketosynthase gene, partial cds	85%; 98%	beta ketosynthase, partial [alpha proteobacterium F16]	88%; 100%	microcystin	Matched with PKSI	–	–	–	–
FF4	*Rhodopirellula* sp.	Not Bioactive	Bioactive	1000 bp	750 bp	–	–	NSR	–	–	NSR	–	–	–	–
FC3	*Rhodopirellula rubra*	Bioactive	Not Bioactive	750 bp	1000 bp	–	–	NSR	–	–	NSR	–	–	–	–
FC17	*Rhodopirellula rubra*	Not assayed	Not assayed	–	1000 bp	–	–	–	–	–	NSR	–	–	–	–
FC15	*Rhodopirellula rubra*	Not assayed	Not assayed	750 bp	1000 bp	–	–	NSR	–	–	NSR	–	–	–	–
MsF5.1	*Rhodopirellula rubra*	Not Bioactive	Not Bioactive	750 bp	–	–	–	NSR	–	–	–	–	–	–	–
OJF1	*Rhodopirellula rubra*	Not assayed	Not assayed	750 bp	1000 bp	*Rhodopirellula baltica* SH 1 complete genome; segment 3/24, dipeptidyl peptidase IV	90%; 100%	–	–	–	*Myxococcus stipitatus* DSM 14675, complete genome, non-ribosomal peptide synthetase	83%; 34%	surfactin synthetase [*Rhodopirellula* sp. SWK7]	94%; 98%	HC-Toxin
LF2	*Rhodopirellula rubra*	Not Bioactive	Not Bioactive	750 bp	–	*Rhodopirellula baltica* SH 1 complete genome; segment 11/24	73%; 68%	putative membrane protein [*Rhodopirellula* sp. SWK7]	97%; 100%	–	–	–	–	–	–
UC9	*Rhodopirellula rubra*	Not Bioactive	Bioactive	750 bp	750 bp	–	–	NSR	–	–	*Planctomyces brasiliensis* DSM 5305, complete genome, prolyl oligopeptidase	68%; 40%	Prolyl endopeptidase [Rhodopirellula sallentiNSR SM41]	98%; 100%	Bacitracin
CcC6	*Rhodopirellula lusitana*	Not Bioactive	Bioactive	600 bp	1000 bp	–	–	NSR	–	–	NSR	–	–	–	–
CcC8	*Rhodopirellula lusitana*	Not Bioactive	Bioactive	600 bp	1000 bp	Alpha proteobacterium F16 beta ketosynthase gene, partial cds	85%; 100%	beta ketosynthase, partial [alpha proteobacterium F16]	88%; 89%	pikromycin	Matched with PKSI	–	–	–	–
FC24	*Rhodopirellula lusitana*	Bioactive	Not Bioactive	750 bp	1000 bp	*Pirellula staleyi* DSM 6068, complete genome	66%; 94%	DUF1501 domain-containing protein [Rhodopirellula sallentiNSR]	91%; 100%	–	NSR	–	–	–	–
FC25	*Rhodopirellula lusitana*	Not Bioactive	Bioactive	750 bp	–	*Pirellula staleyi* DSM 6068, complete genome	66%; 93%	DUF1501 domain-containing protein [Rhodopirellula sallentiNSR]	91%; 100%	–	–	–	–	–	–
FC26	*Rhodopirellula lusitana*	Not Bioactive	Not Bioactive	750 bp	–	–	–	NSR	–	–	–	–	–	–	–
FC27	*Rhodopirellula lusitana*	Not Bioactive	Bioactive	750 bp	–	–	–	NSR	–	–	–	–	–	–	–
SM4	*Rhodopirellula lusitana*	Bioactive	Bioactive	750 bp	1000 bp	–	–	NSR	–	–	NSR	–	–	–	–
UC13	*Rhodopirellula lusitana*	Bioactive	Bioactive	750 bp	1000 bp	–	–	NSR	–	–	*Nocardia cyriacigeorgica* GUH-2 chromosome complete genome, putative non-ribosomal peptide synthetase (modular protein)	72%; 60%	hypothetical protein SMAC_05551 [*Sordaria macrospora* k-hell]	87%; 82%	–
UC16	*Rhodopirellula lusitana*	Not Bioactive	Bioactive	750 bp	1000 bp	–	–	NSR	–	–	NSR	–	–	–	–
UC17	*Rhodopirellula lusitana*	Bioactive	Bioactive	–	–	–	–	–	–	–	–	–	–	–	–
UC20	*Rhodopirellula lusitana*	Not Bioactive	Bioactive	–	–	–	–	–	–	–	–	–	–	–	–
UC22	*Rhodopirellula lusitana*	Not Bioactive	Not Bioactive	750 bp	1000 bp	–	–	NSR	–	–	NSR	–	–	–	–
UC31	*Rhodopirellula lusitana*	Bioactive	Bioactive	750 bp	1000 bp	–	–	NSR	–	–	NSR	–	–	–	–
UC33	*Rhodopirellula lusitana*	Not assayed	Not assayed	750 bp	1000 bp	–	–	NSR	–	–	NSR	–	–	–	–
UC36	*Rhodopirellula lusitana*	Bioactive	Bioactive	600 bp	1000 bp	–	–	NSR	–	–	NSR	–	–	–	–
UC38	*Rhodopirellula lusitana*	Not Bioactive	Not Bioactive	600 bp	1000 bp	Alpha proteobacterium F16 beta ketosynthase gene, partial cds	86%; 100%	beta ketosynthase, partial [alpha proteobacterium F16]	92%; 100%	stigmatellin	Matched with PKSI	–	–	–	–
UC49.2	*Rhodopirellula lusitana*	Not Bioactive	Not Bioactive	600 bp	–	–	–	NSR	–	–	–	–	–	–	–
UF6	*Rhodopirellula lusitana*	Not Bioactive	Bioactive	1000 bp	1000 bp	–	–	NSR	–	–	NSR	–	–	–	–
LF1	*Rubripirellula obstinata*	Not assayed	Not assayed	750 bp	–	–	–	NSR	–	–	–	–	–	–	–
UC8	*Roseimaritima ulvae*	Not Bioactive	Not Bioactive	750 bp	1000 bp	*Chondromyces crocatus* strain Cm c5, complete genome, polyketides synthase	70%; 91%	–	–	myxothiazol	–	–	–	–	–
UF2	*Roseimaritima ulvae*	Bioactive	Not Bioactive	1000 bp	–	*Brassica rapa* subsp. pekinensis clone KBrB081M20, complete sequence	81%; 16%	–	–	–	–	–	–	–	–
UF3	*Roseimaritima ulvae*	Bioactive	Not Bioactive	750 bp	–	*Myxococcus hansupus* strain mixupus, complete genome, Malonyl CoA-acyl carrier protein transacylase	66%; 94%	polyketide synthase ketosynthase domain [*Nostoc* sp. ATCC 53789]	65%; 99%	stigmatellin	–	–	–	–	–
UF4.2	*Roseimaritima ulvae*	Bioactive	Not Bioactive	750 bp	–	*Nonomuraea spiralis* strain IMC A-0156 pyralomicin biosynthetic gene cluster, complete sequence	71%; 59%	–	–	stigmatellin	–	–	–	–	–
FC18	New genus	Not Bioactive	Not Bioactive	–	1000 bp	–	–	–	–	–	–	–	–	–	–
FF15	New genus	Not Bioactive	Not Bioactive	750 bp	850 bp	*Lyngbya majuscula* CCAP 1446/4 clone 7 polyketide sythase gene, partial cds	83%; 100%	polyketide sythase, partial [*Lyngbya majuscula* CCAP 1446/4]	96%; 98%	stigmatellin	NSR	–	–	–	–
Pd1	*Planctomyces* sp.	Not Bioactive	Not Bioactive	600 bp	–	–	–	NSR	–	–	–	–	–	–	–
UiF1 Ent1	*Planctomyces* sp.	Not Bioactive	Not Bioactive	600 bp	850 bp	–	–	NSR	–	–	NSR	–	–	–	–
Gr7	*Planctomyces brasiliensis*	Bioactive	Bioactive	750 bp	–	*Prorocentrum micans* polyketide synthase ketosynthase domain protein gene, partial cds	82%; 99%	polyketide synthase ketosynthase domain protein [*Prorocentrum micans*]	98%; 96%	myxalamid	–	–	–	–	–

*NSR, No Sequence Resulted; Blastn was searched in NCBI using somewhat similar sequences and the selected hit was the one with the highest coverage. Blastp was searched in NCBI using somewhat similar sequences and the selected hit was the one with the highest similarity. NaPDoS is a webserver tool that allows the prediction of biosynthetic pathways*.

The target microorganisms used were grown overnight in 10 mL of Luria Broth (LB) medium, at 37°C and 220 r.p.m. Cultures absorbance was measured at 600 nm and the cell concentration adjusted to 2.5 × 10^5^ CFU/mL.

The extracts were assayed in 96 well-plates in triplicates three times. Ninety microliter of each target culture were incubated with 10 μL of each extract. The cultures were also incubated with 10 μL of the respective positive control: amphotericin B (0.19, 0.39, 0.78, and 1.56 μg.mL^−1^) against *C. albicans*, rifampicin (62.5, 125, 250, and 500 mg.mL^−1^) against *E. coli* and *P. aeruginosa* and chloramphenicol (3.75, 7.5, 15, and 30 μg.mL^−1^) against *B. subtilis* and *S. aureus*. LB medium (100 μL) was used as negative control and 100 μL of the target cultures were used to control the normal growth of the strains, both done in triplicate. To ensure that neither the solvent mixture (acetone+DMSO) nor the media induced inhibition of the cultures, 10 μL of each condition was assayed with 90 μL of culture of each target strain as controls. The inoculated plates were incubated at 37°C and 220 r.p.m for 24 h. Initial and final absorbance measurements were performed at 600 nm in a Multiskan GO plate reader (Thermo Scientific) and analyses of the assays were carried out as described in Graça et al. (2015). Extracts were only considered bioactive when inhibitory values above 20% were obtained in two or more assays showing, thus, consistency in the results.

## Results

The genomic potential of *Planctomycetes* to produce bioactive compounds was assessed by amplification and sequencing of PKS-I and NRPS genes and analysis *in silico* of tree genomes (UC8, LF1, and FC18). Furthermore, screening assays were performed in order to assess the production of antimicrobial molecules.

### PKS-I and NRPS genes, molecular, and *in silico* analysis

The search for the conserved motifs of the ketosynthase of the PKS-I genes and core motif V of NRPS genes was performed in 40 planctomycetes of 10 different taxa (Table 1; Figure S1). Amplicons with several sizes were obtained from 38 strains and bands extracted, DNA purified and sequenced. Only 24 strains were amplified for the expected product size (750 bp) and 10 for other product sizes with the PKS-I primers. With the NRPS primers, 17 planctomycetes amplified for the expected product size (1000 bp) and five for other product sizes (Table 1). Since these genes are understudied in *Planctomycetes*, amplicons of unexpected sizes were also sequenced. It was only possible to retrieve sequences from 18 amplicons. Seven out of the 11 amplicons with 750 bp and 2 amplicons with 600 bp amplified with PKS-I primers confirmed the presence of the ketosynthase gene in the planctomycetes. Two ketosynthase genes were confirmed with the sequencing of NRPS amplicons of 600 bp. Two amplicons of PKS-I genes with 750 bp were found with potential to codify for a DUF1501 domain-containing protein (FC24 and FC25), one for a putative membrane protein (LF2) and another for a *Rhodopirellula* dipeptidyl peptidase IV (OJF1). Concerning the NRPS amplification, only three amplicons provided sequences confirming the presence of NRPS modules (UC13, OJF1, and UC9). The Blast2go tool analysis of the sequenced amplicons confirmed the results obtained in the previous step using Blast in NCBI database (Table S1).

**Figure 1:**
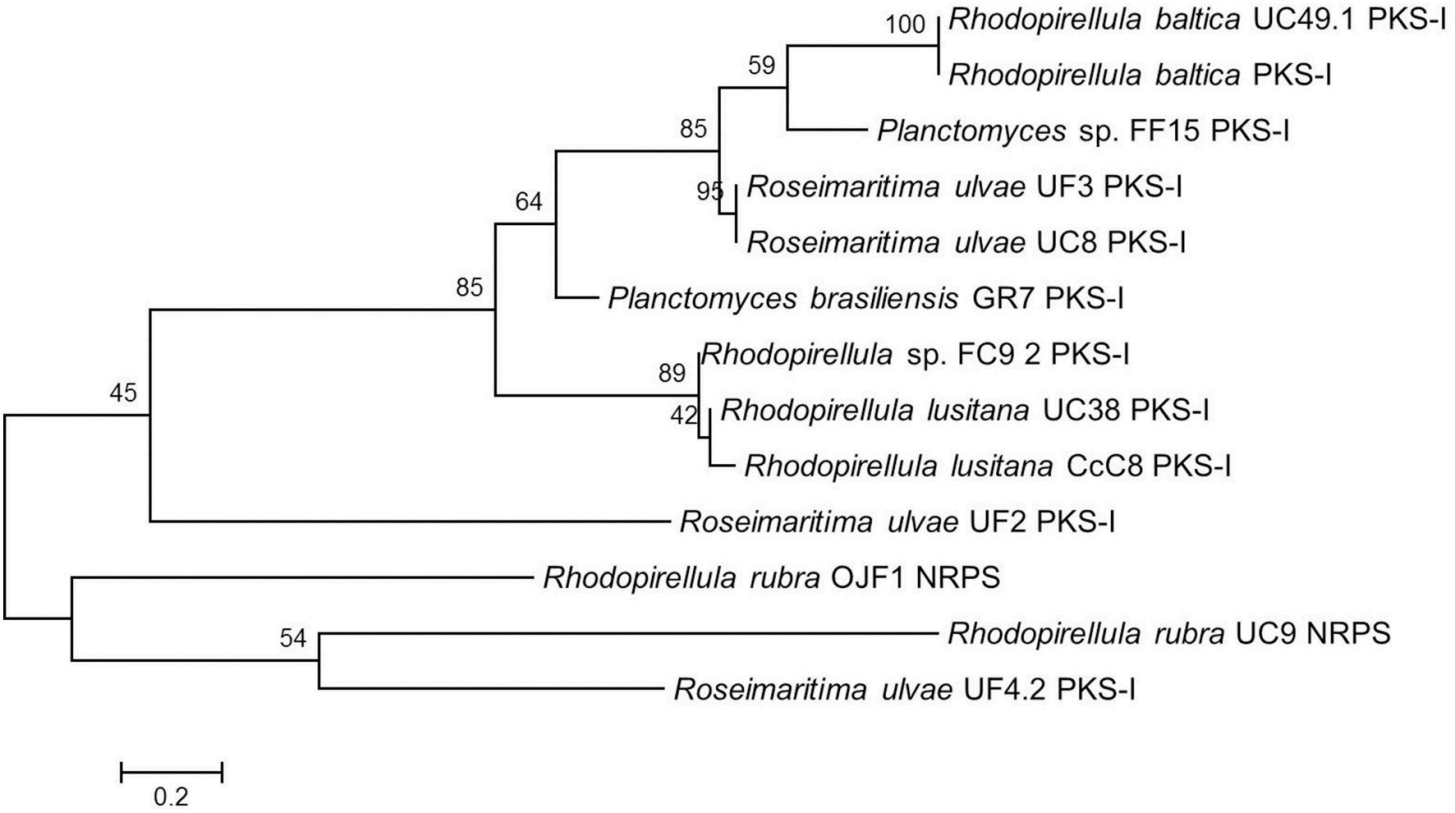
**Maximum Likelihood tree showing the phylogenetic relationships determined from the PKS-I and NRPS gene amino acid translated sequences of the 13 Planctomycetes sequenced amplicons**. Equal input model was used. Bootstrap values were calculated based on 1000 resampling and are shown beside nodes. In general PKS-I tend to group together as well as NRPS. Scale bar = 0.2 substitutions per 100 nucleotides.

The phylogenetic tree using the amino acids corresponding to the amplified PKS-I and the NRPS sequences revealed that the PKS-I clustered together but were separated from the NRPS with the exception of *R. ulvae* UF4.2 PKS-I which may be a hybrid PKSI-NRPS (Figure 1). The PKS-I from two strains of *R. baltica* clustered together as well as those of *Rhodopirellula lusitana*. When considering the site sampling, phylogeny (Figure S1), or macroalgal host-origin of the planctomycetes analyzed (Lage and Bondoso, 2011), no correlation could be obtained.

NaPDoS analysis showed that 10 of the amplified genes encoded for modular KS and have somewhat similarity with known secondary metabolite pathways involved in the production of antitumor (UC49.1, UC8, and Rb), antibiotic (Gr7 and CcC8), antifungal (Gr7), quinol inhibitor (FF15, UF4.2, UF3, and UC38), and toxins (FC9.2) (Table 1). Regarding NRPS genes, NaPDoS *in silico* analysis showed a codification of C domain with potential production of a toxin for OJF1 and a DCL domain with potential production of bacitracin for UC9. Since the percentage of similarity of the sequences obtained using the NaPDoS is less than 80%, the prediction had to be confirmed by blastnr. Furthermore, *R. ulvae* strains most probably have uncharacterized biosynthetic gene clusters since similarities lower than 85% were obtained with blastnr. In fact, comparing the different analyses *in silico* performed, all the strains belonging to the taxa *R. ulvae* (UC8, UF3, and UF4.2) showed the presence of a PKS-I gene cluster not yet characterized being the highest percentage of similarity of 71% in strain UF4.2.

### Secondary metabolite related *in silico* analysis of UC8, FC18, and LF1 genomes

**Figure 2:**
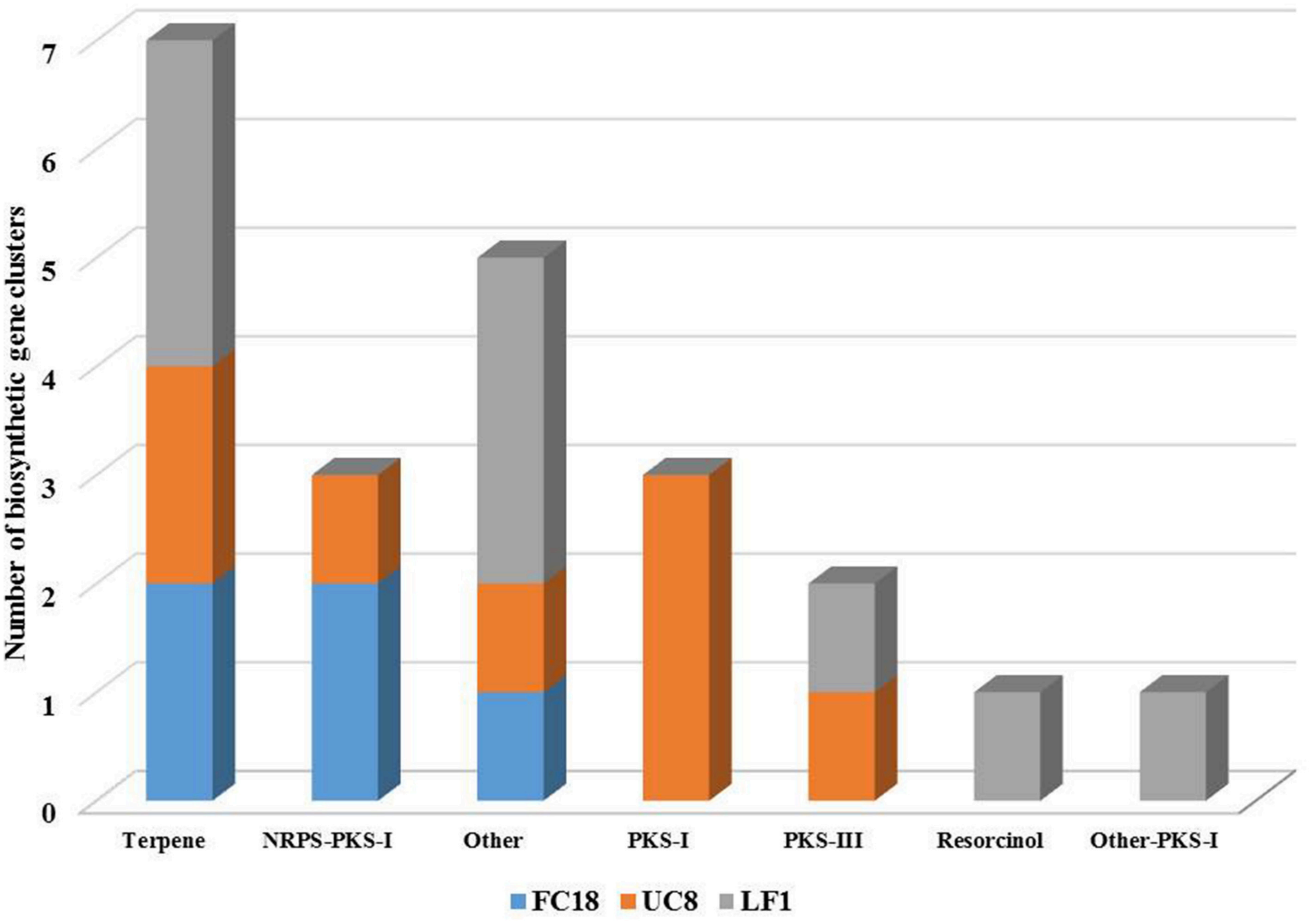
**Numerical comparison of biosynthetic gene clusters present in genomes of strains LF1, UC8, and FC18 using antiSMASH analysis tool**. Strains LF1 and UC8 are comparatively the ones with a higher number of biosynthetic gene clusters. LF1 lacks NRPS genes.

Nowadays, several are the tools available to search for biosynthetic clusters, which are able to predict and provide information on the genomic potential for the production of bioactive compounds. In this work, antiSMASH, and NaPDoS tools were used. The antiSMASH analysis showed that LF1 was the one with higher number of biosynthetic clusters (9), followed by UC8 (8), and FC18 (5; Figure 2). UC8 is the only strain that possesses PKS-I clusters and LF1 the only one that possesses a resorcinol cluster.

**Figure 3:**
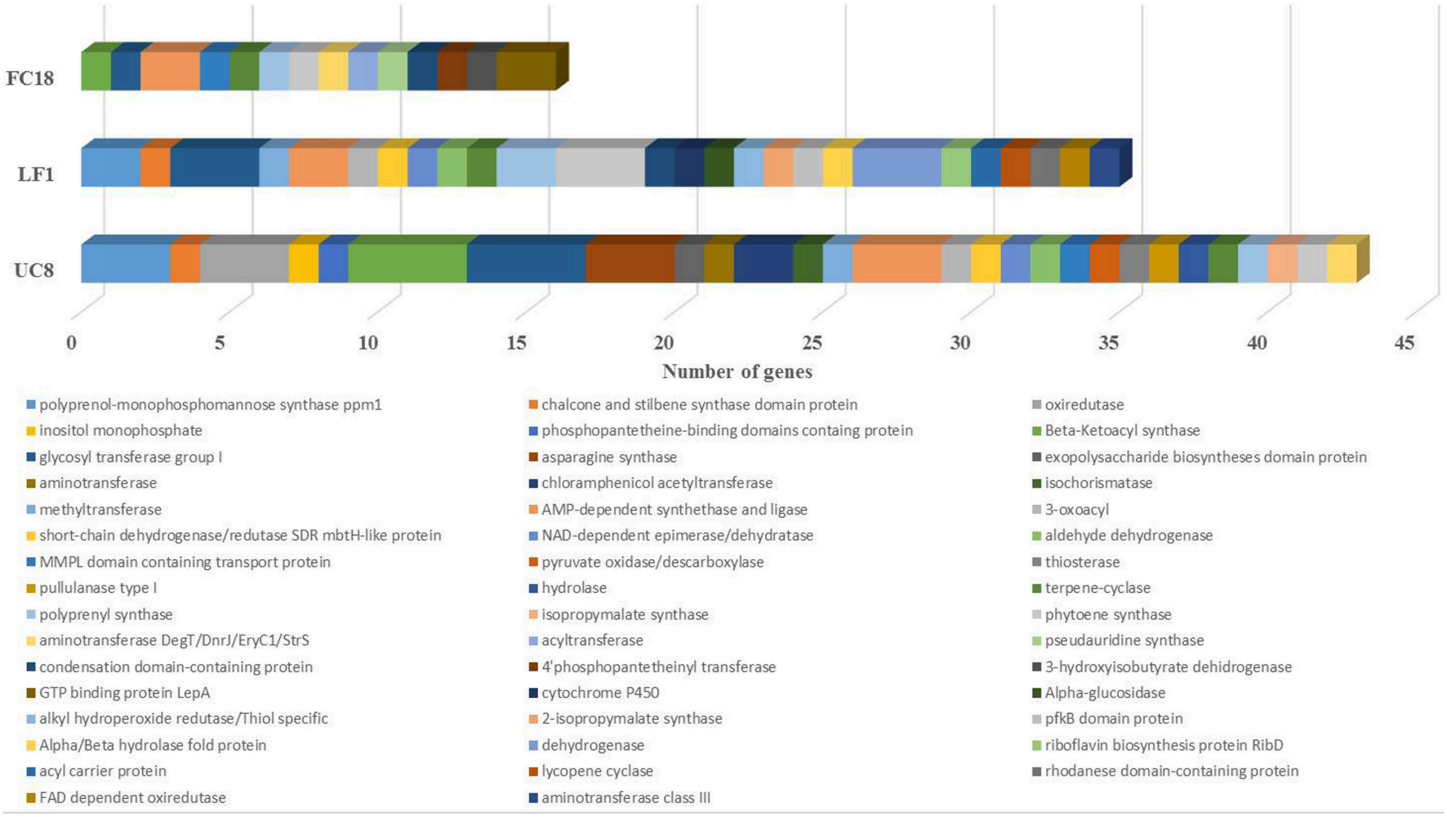
**Comparison between the number of biosynthetic genes present in strains LF1, UC8, and FC18 genomes using antiSMASH analysis**. UC8 followed by LF1 have more diverse biosynthetic genes. Common to the 3 strains are glycosyl transferase group I, AMP-dependent synthethase and ligase, terpene-cyclase, polyprenyl synthase, and phytoene synthase genes.

However, a closer analysis of the clusters related to secondary metabolites revealed that UC8 is the strain with higher number of biosynthetic genes (43), followed by LF1 (35), and FC18 (16; Figure 3). The genes codifying for glycosyl transferase group I, AMP-dependent synthethase, and ligase, terpene-cyclase, polyprenyl synthase, and phytoene synthase are shared by all the strains. Additionally to these genes, UC8, and LF1 are the strains that share more genes (7), while UC8 and FC18 share 3 genes, and LF1 and FC18 only one. Regarding unique biosynthetic genes, UC8 is again the one that possesses the higher number with 18 genes, LF1 has 15 genes, and FC18 only 6 (Figure 3).

Although LF1 is the strain with the higher number of biosynthetic clusters it does not have any kind of NRPS gene (Figure 2) and contains the lowest number of regulatory genes (Figure S2). LF1's genome also contains in one cluster a codification for an unknown biosynthetic pathway with a regulatory gene similar to a *Lux*R response regulator and another cluster coding for a terpene which has an *Ara*C family transcriptional regulator and two sigma-54 dependent transcriptional regulators. One cluster of UC8 is a hybrid NRPS-PKS-I which encodes for an epothilone biosynthetic gene and holds a *tetR* family transcriptional regulator. A PKS-I cluster of UC8 holds a *Gnt*R family transcriptional regulator. There are still five clusters with unknown attributed function and in all the clusters there are still some uncharacterized biosynthetic, transport and regulatory genes (Figure 2; Figure S2).

**Figure 4:**
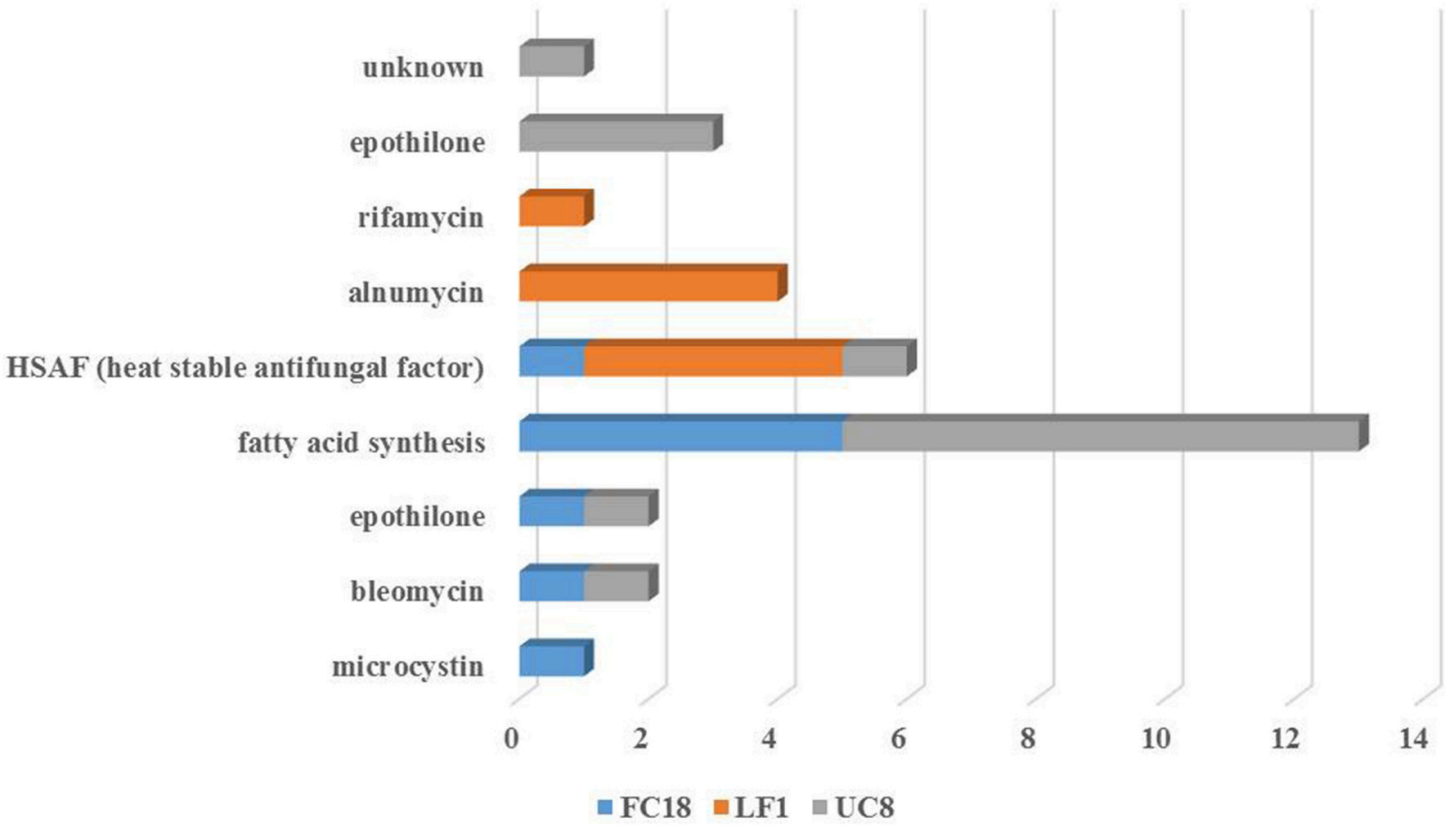
**Pathway product prediction of the strains LF1, UC8, and FC18 genomes analysis using NaPDoS tool**. Anticancer, antibiotic and antifungal are the potential repertoire evidenced by the analyses of these genomes.

NaPDoS analysis of genomes secondary pathways confirmed (1) gene similarities lower than 80%; (2) lack of any kind of NRPS genes in LF1; (3) the presence of NRPS-PKS hybrids in FC18 and UC8; and (4) that UC8 was the strain with the highest number of secondary metabolite genes (Figure 4). Hepatoxic (FC18), anticancer (FC18 and UC8), antifungal (LF1, UC8, and FC18), and antibiotic (LF1) activities were predicted by NaPDoS.

### Bioactivity assays with planctomycetes

The production of bioactive compounds by the planctomycetes was only assessed in 35 strains due to growth limitation (Table 1). Two culture media (M600 and M607) and four types of extracts were assayed. Forty-three percent (15 strains) of the strains were active against *C. albicans* and 54% (19 strains) against *Bacillus subtilis*. Low activity was detected against the two strains of *E. coli* and no activity was obtained against *P. aeruginosa* and *S. aureus*.

**Figure 5:**
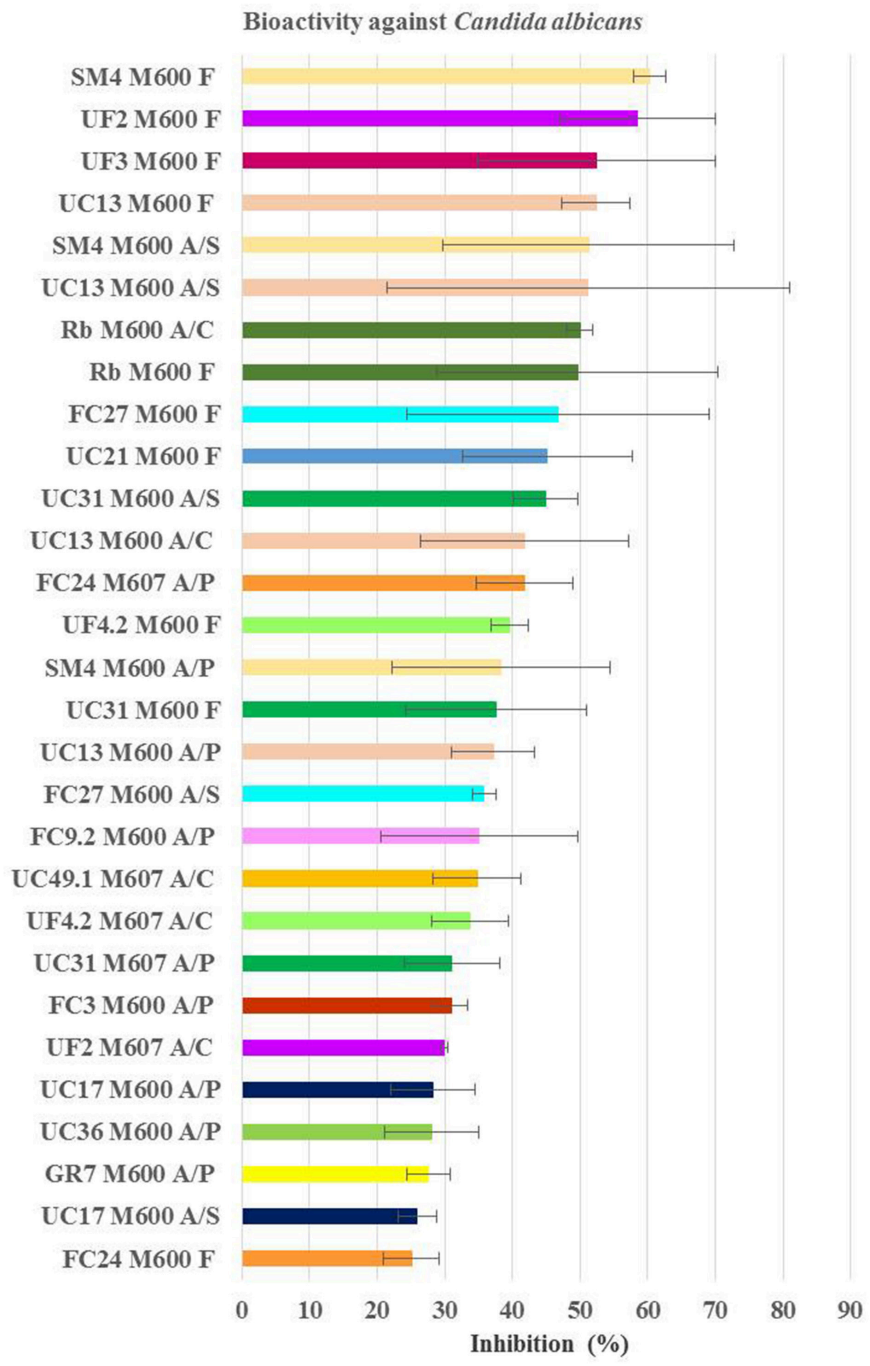
**Average inhibition (percentage) of the positive extracts against *Candida albicans* obtained from planctomycetes**. Medium M600 was the one providing more extract bioactive against *C. albicans* and higher inhibition values were obtained from the filtrate extract. M600 and M607—medium of bacteria incubation; A/S—extract with acetone+DMSO from the supernatant; A/P—extract with acetone+DMSO from the pellet; A/C—extract with acetone+DMSO from the culture broth; F—extract from filtrate of the supernatant.

Figure 5 shows planctomycetes inhibition obtained against *C. albicans*. The two more relevant extracts due to consistency and level of inhibition were the aqueous supernatants of the culture broths (F) in M600 from SM4 (average activity of 60%) and from UC13 (average activity of 52%). The majority of the bioactive extracts against *C. albicans* are from bacteria incubated in medium M600 (83%). UC13 was the only strain that induced inhibition with all extracts from M600. Three extracts were bioactive for SM4: pellet (A/P), organic supernatant (A/S), and aqueous supernatant (F). Only the supernatant extracts (A/S and F) from the strains UC31, FC27 when incubated in M600 were active. For *R. baltica* SH1^T^ only extracts A/C and F in M600 were active. Strains FC3, FC9.2, UC36, and Gr7 only induced bioactivity when the extracts were obtained from the pellet. Strains UF2 and UF4.2 induced bioactivity with the aqueous supernatant (F) when incubated in M600. However, when these strains were incubated in M607 the bioactive extract was obtained from the organic crude extract.

**Figure 6:**
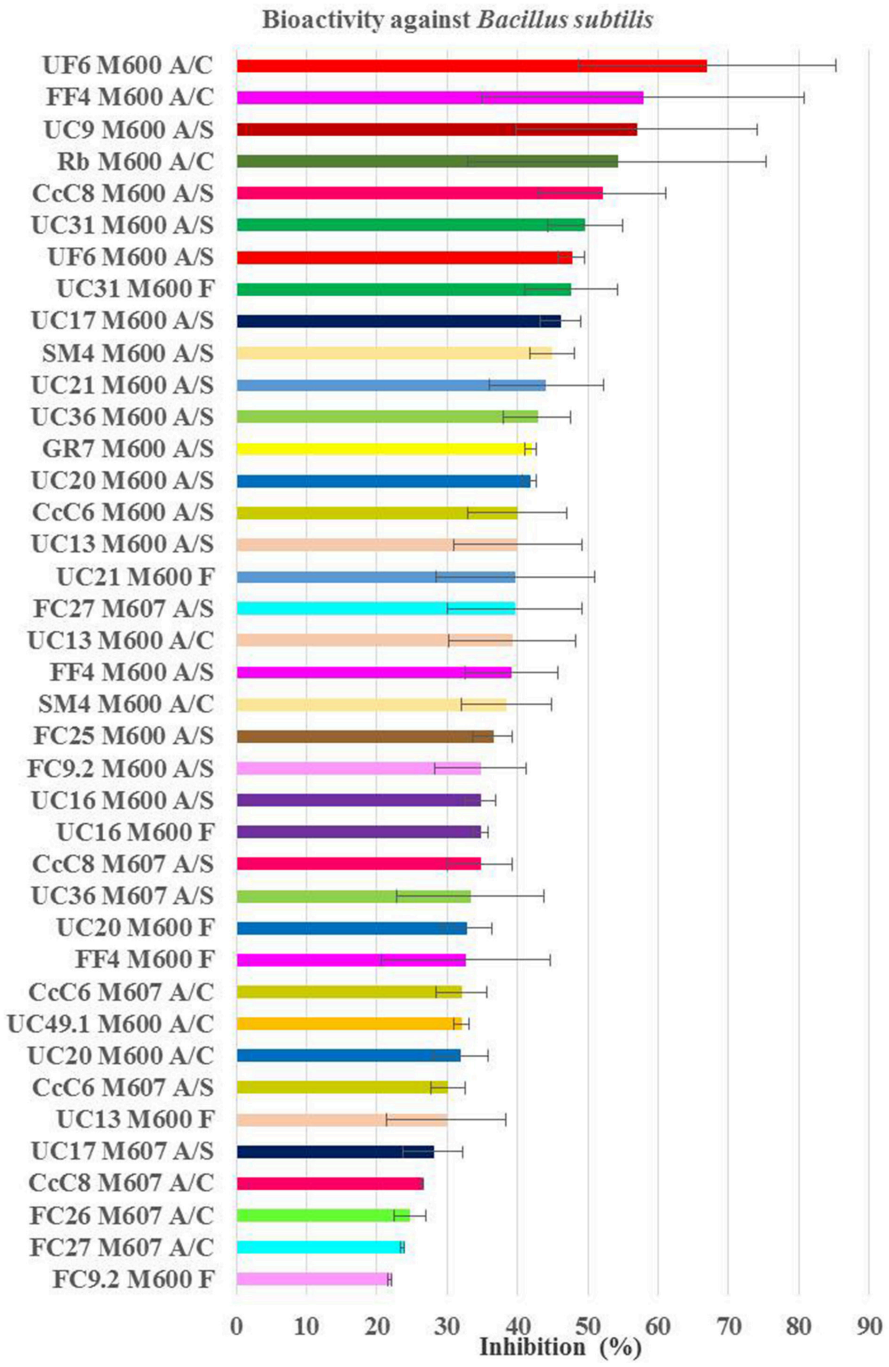
**Average inhibition (percentage) of the positive extracts against *Bacillus subtilis* obtained from planctomycetes**. Medium M600 was the one providing more extract bioactive against *B. subitilis* and higher inhibition values were obtained from the organic extracts. M600 and M607—medium of bacteria incubation; A/S—extract with acetone+DMSO from the supernatant; A/P—extract with acetone+DMSO from the pellet; A/C—extract with acetone+DMSO from the all culture broth; F—extract from filtrate of the supernatant.

Regarding *Bacillus subtilis* (Figure 6), the strains with consistent higher values of inhibitions were the organic supernatants (A/S) in M600 of CcC8, UC31, and UF6 (average activity of 52, 50, and 48%, respectively). However, the crude extract of UF6 in M600 was the most inhibitory one with 66% of average inhibition. The nine extracts with higher activity against *B. subtilis* had approximately the same inhibitory activity as 7.5 μg/ml chloramphenicol (the third concentration of the MIC). The extracts obtained from strains incubated in M600 (77% strains) were more active than the ones incubated in M607. Sixty-eight percent of the bioactive extracts were obtained from the supernatant of the cultures broths [organic (A/S) or aqueous (F)]. No pellet extract was active. While F extract in M600 obtained from FC9.2 was the least inhibitory (22% inhibition), the value of inhibition raised to 35% when the supernatant was extracted with acetone. The bioactive extracts of CcC6 in M607, CcC8 in M607, FC27 in M607, SM4 in M600, and UF6 in M600 were A/C and A/S which may indicate that the bioactive molecules are possibly polar and secreted to the media. Also FF4, UC13, and UC20 may produce bioactive molecules to the media since all the bioactive extracts were obtained from A/S and F extracts.

Ten strains were able to produce both antibiotic and antifungal compounds: *R. baltica* SH1^T^; UC49.1; UC21; SM4; UC13; UC17; UC31; UC36, Gr7; and FC9.2. In this work, 24 planctomycetes (69%) revealed bioactivity potential.

## Discussion

Nonribosomal peptides and polyketides are important groups of secondary metabolites accounting for a significant portion of known natural products (Walsh, 2004; Wang et al., 2014). In an extensive genome mining of NRPS and PKS genes across the three domains, Wang et al. (2014) observed that NRPS and type I PKS clusters were more frequent in the phyla *Proteobacteria, Firmicutes, Actinobacteria*, and *Cyanobacteria*. Regarding *Planctomycetes* only a significant lower number (seven) was analyzed of which five possessed PKS and hybrid NRPS/PKS clusters. These results are in accordance with ours since we only detected PKS and hybrids genes.

In our screening, although a high number of amplicons was obtained for both type I PKS (66%) and NRPS (47%) genes, only a reduced number could be confirmed to be PKS or NRPS genes after sequencing. This may be due to the presence of more than one KS and NRPS domain which may interfere with the direct sequencing of the amplicons. Furthermore, four non-specific amplifications (LF2, FC24, FC25, and UF2) were also obtained with these primers which indicate the necessity to confirm the amplifications obtained by sequencing. The phylogenetic analyses of the retrieved PKS-I and NRPS sequences also suggested that some planctomycetes that clustered together (Figure 1; FC9.2, UC38, CcC8) may possess hybrid NRPS/PKS-I modules because the amplified PKS-I sequences (Table 1) were obtained with NRPS primers. The detection of PKS genes with NRPS primers may also be a result of the low specificity of the degenerate primers used in our work. Tambadou et al. (2014) also obtained sequences without similarity to the A domain of NRPSs in their study with marine mudflat bacteria.

The molecular analysis performed (Table 1) confirmed the previous genome mining result obtained for *R. baltica* SH1^T^ which showed the presence of a type I PKS gene in this bacterium (Jeske et al., 2013). Furthermore, the NaPDos tool predicted that this gene, present in both *R. baltica* strains SH1 and UC49.1, may encode for an epothilone. Gene structural similarity between the two *R. baltica* is also evidenced by their phylogenetic relationship (Figure 1). Although no absolute confirmation can be taken regarding putative products, the analysis of the closest similar products indicates an epothilone pathway for the two strains of *R. baltica*. Epothilones are a new class of tubulin target agents effective against human malignant disease (Cheng et al., 2008). Originally epothilone was discovered from *Sorangium cellulosum*, a myxobacterium isolated from the banks of Zambesi River in Africa, and showed to be highly cytotoxic *in vitro* to the human T-24 bladder carcinoma cell line (Gerth et al., 1996).

The presence of a polyketide synthase gene in *Planctomyces* strain Gr7 is shared with *Planctomyces brasiliensis* (Jeske et al., 2013) and these two planctomycetes have a 16 S rRNA gene similarity of 100% (Lage and Bondoso, 2011) which demonstrates their closeness. A predicted myxalamid pathway was foreseen for the type I PKS gene sequenced from *Planctomyces* strain Gr7. Myxalamids are antibiotics produced by the myxobacterium *Myxococcus xanthus* and are known to block the respiration chain at the site of complex I, i.e., NADH:ubiquinone oxidoreductase (Gerth et al., 1983).

*R. lusitana* strain UC38, *R. ulvae* UF3, and UF4.2 and an uncharacterized new genus, strain FF15, revealed to amplify a type I PKS gene with a predicted production of stigmatellin which is an antibiotic produced by the myxobacterium *Stigmatella aurantiaca* (Oettmeier et al., 1985). This antibiotic is a potent electron transfer inhibitor of photosynthetic and respiratory electron transports, inhibiting the quinol oxidation (Qo) site of the cytochrome b6f complex of thylakoid membranes, of the cytochrome bc1 complex (ubiquinol-cytochrome c reductase or complex III) in mitochondria and of the bacterial photosynthetic reaction center (Thierbach et al., 1984; Giangiacomo et al., 1987).

*R. ulvae* UC8 amplified also for a type I PKS gene that potentially encodes for a myxothiazol which is an antifungal antibiotic from the myxobacterium *Myxococcus fulvus* (Gerth et al., 1980). Although its binding site is different of that of stigmatellin, myxothiazol also is a competitive inhibitor of ubiquinol. It binds at the quinol oxidation (Qo) site of the mitochondrial cytochrome bc1 complex (Ouchane et al., 2002). On the original screening of myxothiazol, Gerth et al. (1980) observed growth inhibition of *B. subtilis* in plate culture although no MIC concentration was determined. However, good effectiveness of this antibiotic was detected against filamentous fungi (Gerth et al., 1980). In our study no bioactivity was detected by *R. ulvae* UC8 against neither *C. albicans* nor *B. subtilis* so optimization of growth conditions is needed in order to attempt the induction of such synthetic pathway.

Our results suggest that *Planctomycetes* potentially may have several similar secondary metabolite pathways (epothilone, stigmatellin, myxothiazol, myxamid) to *Myxobacteria*. This bacterial group is known for their complex cell cycle, bioactive potential, and large genomes (Schneiker et al., 2007; Huntley et al., 2012). Also the gene synteny analysis revealed higher similarity with *Myxobacteria* PKS-I genes than with other *Planctomycetes* (Figure S3).

Besides secondary pathways related to *Myxobacteria*, other pathways are also present. Microcystin pathway is possibly encoded by *R. lusitana* CcC8 and *Rhodopirellula* sp. FC9.2. The synteny analysis of the PKS-I amplicon from FC9.2 revealed its similarity to mcyE gene from *Mycrocystis aeruginosa* NIES 843 (Figure S3). Microcystins are a class of cyclic heptapeptide toxins produced by several freshwater cyanobacteria namely members of *Microcystis, Planktothrix, Anabaena, Oscillatoria*, and *Nostoc* but recent evidence also suggest that they are also being produced in the oceans by a number of cosmopolitan marine species (Vareli et al., 2013). Microcystin biosynthesis gene cluster has a highly conserved organization which includes NRPS, PKS, and hybrid NRPS-PKS genes as has been characterized in the genus *Microcystis* (Noguchi et al., 2009).

Regarding NRPS, UC9 demonstrated putative capacity for the production of bacitracin, a mixture of cyclic peptides with antibiotic properties through the inhibition of the cell wall synthesis, produced by strains of *Bacillus licheniformis* (Stone and Strominger, 1971). However, when gene synteny of the NRPS was analyzed, closer similarity was obtained with genes from Planctomycetes and with a different strain of *Bacillus* (Figure S3) Furthermore, *R. rubra* OJF1 NRPS matches a HC-toxin that is the host-selective toxin of the maize pathogen (Walton, 2006).

Noteworthy is the low similarity of the predicted pathways obtained in the analysis with NaPDoS tool databases which can reveal the undiscovered pathways possessed by these strains. The blastnr PKS-I and NRPS values obtained are indicative of new secondary pathways, especially the PKS-I amplified from *R. ulvae* strains which have the lowest similarity to their closest relatives. Although the other planctomycetes strains used in this study possess higher similarity values, their closest relatives do not have their pathways characterized.

In the genome analysis of the three planctomycetes (LF1, FC18, and UC8), besides PKS and NRPS clusters, a resorcinol cluster was found in LF1. Resorcinol can be used as an antiseptic and disinfectant in the treatment of chronic skin diseases such as psoriasis, hidradenitis suppurativa, and eczema, as an oxidative hair coloring product and as a food additive [SCCS (Scientific Committee on Consumer Safety), 2010]. Furthermore, terpene clusters are present in the three genomes. Terpenes comprehend the largest class of plant natural products (Boutanaev et al., 2015) but their bacterial origin has been known for many years and terpene synthases are widely distributed in bacteria (Yamada et al., 2015). Terpenes seem to be present in all planctomycetes genomes (Jeske et al., 2013; Aghnatios et al., 2015) and terpenoid synthases may be likely involved in carotenoid synthesis (Jeske et al., 2013).

Strain LF1 has a transcriptional regulator of the AraC family associated with the cluster coding for a terpene. These regulators are widespread among bacteria and may be involved in the stress responses to virulence (Frota et al., 2004). LF1 also has extracytoplasmic function sigma factor (ECFs), two sigma-54 dependent transcription regulators. This particular ECF has been regulated to various cellular processes like mobility, virulence, biofilm formation, and nitrogen assimilation (Francke et al., 2011). Although different ECFs were found, their presence in planctomycetes has been previously described (Jogler et al., 2012). Through the study of transcriptional regulators and transporter genes found associated to the biosynthetic clusters, the production of the secondary metabolites may be optimized as well as their signaling induction.

The genomes analyzed sustain the relation between large genomes and high number of biosynthetic clusters (Jeske et al., 2013). However, FC18 is the strain with lower number of biosynthetic clusters and genes which could be related to the fact that it was the one that also provided the lowest information for the annotation analysis. Also the absence of NRPS genes in LF1 is in line with Jeske et al. (2013) where, summing up all the results, only 38% of the planctomycetes possess any type of NRPS genes. The secondary metabolite potential of our strains falls short of that of *Gemmata massiliana* and *Schlesneria paludicola* which are still the strains with the highest number of secondary metabolites pathways (Aghnatios et al., 2015)

The products obtained by pathway prediction of the three genomes using NaPDoS showed that these strains are potentially able to produce a heat-stable antifungal factor (Figure 4). This novel model of antifungal activity is encoded by a hybrid NRPS-PKS that induces inhibition against a wide range of fungal species (Yu et al., 2007). Furthermore, UC8 and FC18 showed genomic potential to produce anticancer agents due to the potential presence of epothilone and bleomycin pathways. Bleomycin is a polyamine antibiotic produced by *Streptomyces verticillus* known for its use in cancer treatment and also its toxicity to bacteria and some tissues (Cohen and Josephine, 1976). Strain LF1 possesses genomic potential for the production of antibiotics with predicted pathways for rifamycin and alnumycin that can show different bioactive properties. Ryfamycin is an important antibiotic produced by several bacterial strains like *Nocardia* RSP-3, *Amycolatopsis mediterranei*, marine *Actinobacteria* and the marine sponge bacteria *Salinispora* and it is used to treat tuberculosis leprosy and AIDS-related mycobacterial infections (El-Tayeb et al., 2004; Kim et al., 2006; Hewavitharana et al., 2007; Mahalaxmi et al., 2008). Alnumycin, initially discovered from an endophytic *Streptomyces* sp., is an antibiotic with several biological activities like inhibition of Gram positive bacteria and human leukemia cells (Bieber et al., 1998).

Merging the information from the molecular and screening assays we were able to confirm the genomic potential of nine strains (SH1, UC49.1, FC9.2, UC9, CcC8, UC13, UF3, UF4.2, Gr7). However, for 3 strains (UC38, UC8 and FF15), their bioactivity is still undisclosed. This may be due to the specificity of the produced compounds that may target different strains or possess activities not screened for, such as antitumor or antimalarial, or even to the need of different induction conditions for the production of the compounds.

Stepping to the antimicrobial production, *R. baltica* showed antibiotic and antifungal capacity. *R. lusitana* was the species with the highest values of bioactivity against both *C. albicans* and *B. subtilis*. Although *R. rubra* amplified for PKS-I and NRPS genes no secondary metabolite pathway could be predicted with NaPDoS and only bioactivity was detected against *B. subtilis* by one strain (UC9). *R. ulvae* putatively possesses genes encoding for the production of the antibiotics myxothiazol and stigmatellin and demonstrated high level of activity against *C. albicans* with the filtrate extracts of UF2, UF3, and UF4.2, lower activity with organic crude (A/C) extracts and no activity against *B. subitlis*. The novel genus of strain FF15 showed putative synthetic capacity for the production of stigmatellin but no bioactivity could be detected. *P. brasiliensis* Gr7 demonstrated a great antimicrobial capacity and the genomic potential production of myxalamid.

Regarding the bioactivity screening, while some pellet extracts were bioactive against *C. albicans*, none was active against *B. subtilis* showing that the antifungal compounds were released to the external medium. This is reinforced by the fact that most of the bioactive extracts were obtained from the supernatants (F). The fact that the number of bioactive supernatant extracts obtained with acetone is lower than the aqueous ones can be explained by low affinity of the molecules with the organic solvent used. Occasionally, the pellet and supernatant extracts obtained from a strain were both active while the crude was not, suggesting, thus, an effect among the molecules extracted. This effect may be due to interference with binding sites or counter active actions of the molecules (Jia et al., 2009).

Bioactivity against *C. albicans* and *B. subtilis* was mainly obtained when the planctomycetes were grown in M600 (respectively, 83 and 77% of the extracts), which is, comparatively to M607, a medium with four fold yeast extract, peptone and glucose. M600 appears to be the best medium for bioactivity screenings of planctomycetes. It seems to favor their antimicrobial production due to the favorable higher organic medium conditions and/or the higher biomass yield (absorbance measurements obtained when the bacteria were grown with M600 were in general twice higher than the ones obtained when incubated with M607—data not shown). These favor the growth of fast growing heterotrophs which compete against slower growing microorganisms like planctomycetes (doubling times of several hours to days). The complexity of species and interactions in biofilms impose the development of survival strategies for their members. Moreover, all strains were isolated from the surface of macroalgae, where they are subjected to high levels of released polysaccharides and various forms of environmental stresses like abrupt and broad changes in salinity and temperature, high light intensities, and pollution. As antimicrobials production was higher under higher organic medium conditions, we postulate that high levels of organic carbon may favorably trigger the production of antimicrobials by planctomycetes, a plus for bacteria fighting for their presence in biofilms. Several compounds, mainly polysaccharides, released by macroalgae were identified as potential triggers for secondary metabolite production (Jeske et al., 2013). *In vitro* antibiotics production is commonly favored by starvation conditions which are opposite to the hypothesis here formulated. However, we do not know antibiotics role in the natural environment and how they favor the producing organisms, but it is believed that they control other microbes competing with their neighbors for space and resources (Clardy et al., 2009; Hibbing et al., 2010).

Our results provide diverse and consistent evidence of secondary metabolite production by several planctomycetes with the production of putative novel metabolites of biotechnological interest. Furthermore, the study of planctomycetes compounds that are already known is of the utmost importance as they may have different properties, namely regarding side effects. The presence of genes related to bioactive pathways or bioactivity production in all strains here studied seem to be indicative of their potential ability to fight against their competitor in the biofilm of macroalgae. The analysis of the three genomes (FC18, LF1, and UC8) also revealed that these strains possess a great potential to cope with environmental stress (unpublished results). We should emphasize that this is the first study reporting a high percentage of Planctomycetes extracts with a great antibiotic and antifungal activity against *Bacillus subtilis* and *C. albicans*. Further, studies into the secondary molecules production by these scarcely studied bacteria open the door to new promising and challenging biotechnological studies.

## Author contributions

Conceived and designed the experiments: AG, OL. Performed the experiments: AG, RC. Analyzed the results: AG, RC, OL Wrote the manuscript: AG, RC, OL. All authors have read and approved the manuscript.

## Funding

This research was partially supported by the Strategic Funding UID/Multi/04423/2013 through national funds provided by FCT—Foundation for Science and Technology and European Regional Development Fund (ERDF), in the framework of the programme PT2020 and by the Structured Program of R&D&I INNOVMAR – Innovation and Sustainability in the Management and Exploitation of Marine Resources (reference NORTE-01-0145-FEDER-000035, Research Line NOVELMAR), funded by the Northern Regional Operational Programme (NORTE2020) through the European Regional Development Fund (ERDF).

### Conflict of interest statement

The authors declare that the research was conducted in the absence of any commercial or financial relationships that could be construed as a potential conflict of interest.

## Supplementary material

Annex 1 **PKS-I sequences not used in the phylogenetic tree because of their small bp size and not submitted to GenBank database**.

Table S1 **Results of the *in silico* analysis performed with the Blast2go tool**. These results confirm the ones obtained manually by search with Blastnr in GenBank database.

Figure S1 **Phylogenetic 16 S rDNA tree generated by maximum-likelihood analysis based in General Time Reversible model and Gamma distributed with Invariant sites (G+I) indicating the relationship of the *Planctomycetes* used in this work**. Bar—0.05 substitutions per 1000 nucleotides.

Figure S2 **Number and description of transport and regulatory genes present in strains LF1, UC8, and FC18 genomes reported by antiSMASH analysis**. UC8 is the strain with higher number of regulatory genes.

Figure S3 **Result of the synteny analysis of *Planctomycetes* PKS and NRPS amplicons sequences with the closest strains as matched in NaPDoS**. All the strains were also searched against *Planctomycetales* group for synteny. Only the results with higher similarity are shown.
