# Supplementary material for: Planctomycetes as Novel Source of Bioactive Molecules
Source: Front Microbiol. 2016 Aug 12;7:1241. doi: 10.3389/fmicb.2016.01241 (PMC4982196; doi:10.3389/fmicb.2016.01241)
Supplement: Annex 1 — PKS-I sequences not used in the phylogenetic tree because of their small bp size and not submitted to GenBank database. [file DataSheet1.PDF]

>UF4.2 PKS-I

ATCATTGCCCAAACAAAAGACCGGCATGTTCGAGGTATCAGCGGGAACGACTACGTGCGACTGCTGGC
CCAGAACGGCGACCCCGCCGCTCTGGACGCTTACCTGGGCGTTGGCAACGCACTCAGTATCGCCGCCG
GTCGGTTGTCGTTCTTCCTTGGTATCCAAGGCCCCACAATGGCGCTGGATAC

>OJF1 PKS-I

AACCTTGGACAACGCATCTCGGTTTTCGGTGACCAATCCCATGTAGCGTTCTTGGTAGATCGTGTCGTA
ATCCAGCTGGTCCGGAACGGGGGCGACCGCGATTCCCATCGAATACTGCTCGGGGA
