# Supplementary figures and images for: Planctomycetes as Novel Source of Bioactive Molecules
Source: Front Microbiol. 2016 Aug 12;7:1241. doi: 10.3389/fmicb.2016.01241 (PMC4982196; doi:10.3389/fmicb.2016.01241)

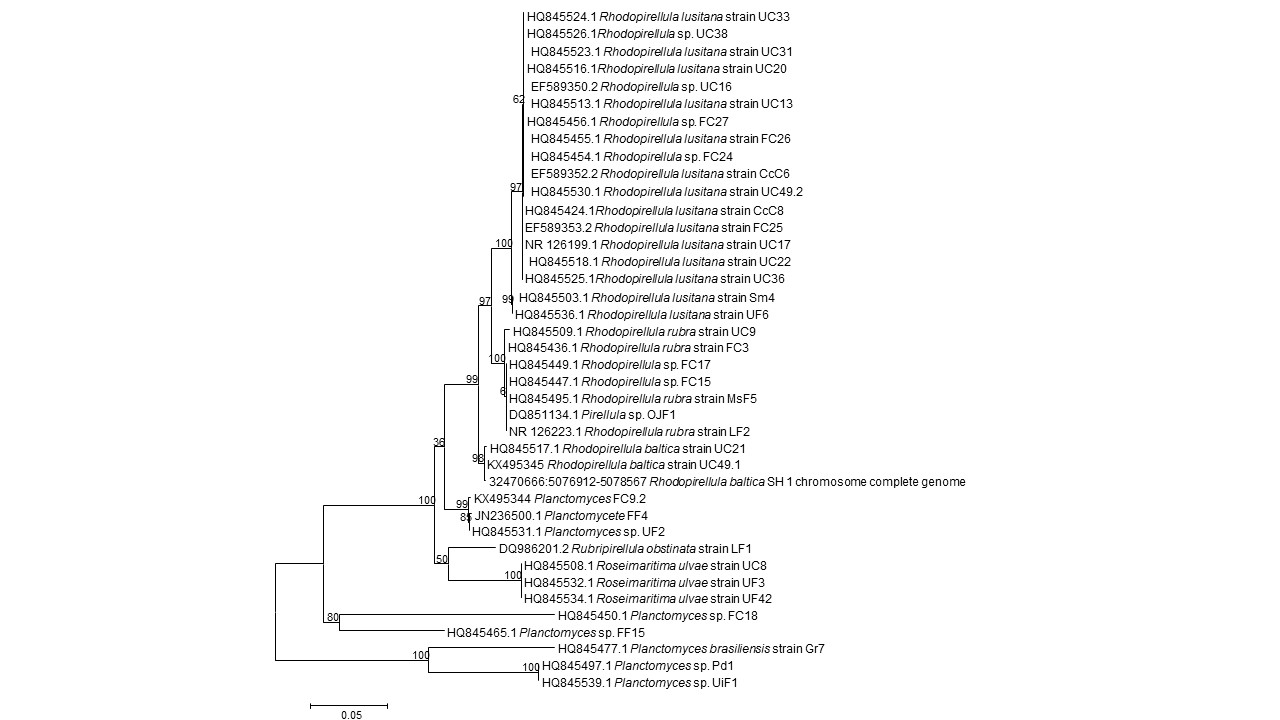

Supplement: Figure S1 — Phylogenetic 16 S rDNA tree generated by maximum-likelihood analysis based in General Time Reversible model and Gamma distributed with Invariant sites (G+I) indicating the relationship of the Planctomycetes used in this work. Bar—0.05 substitutions per 1000 nucleotides. [file Image1.JPEG]

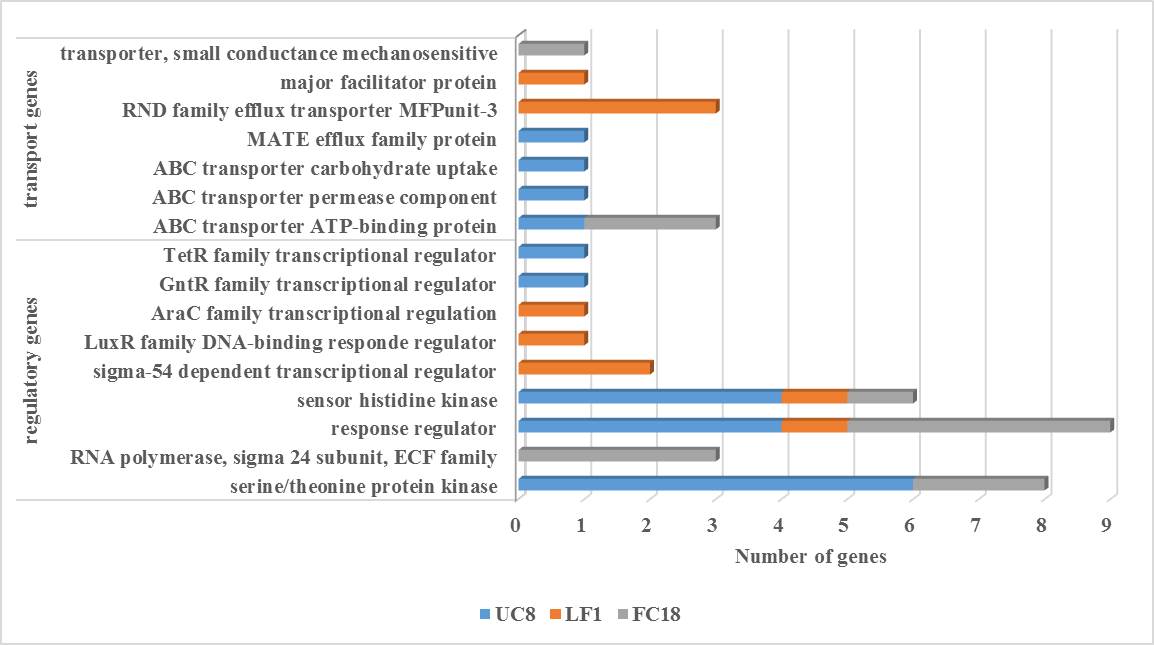

Supplement: Figure S2 — Number and description of transport and regulatory genes present in strains LF1, UC8, and FC18 genomes reported by antiSMASH analysis. UC8 is the strain with higher number of regulatory genes. [file Image2.JPEG]

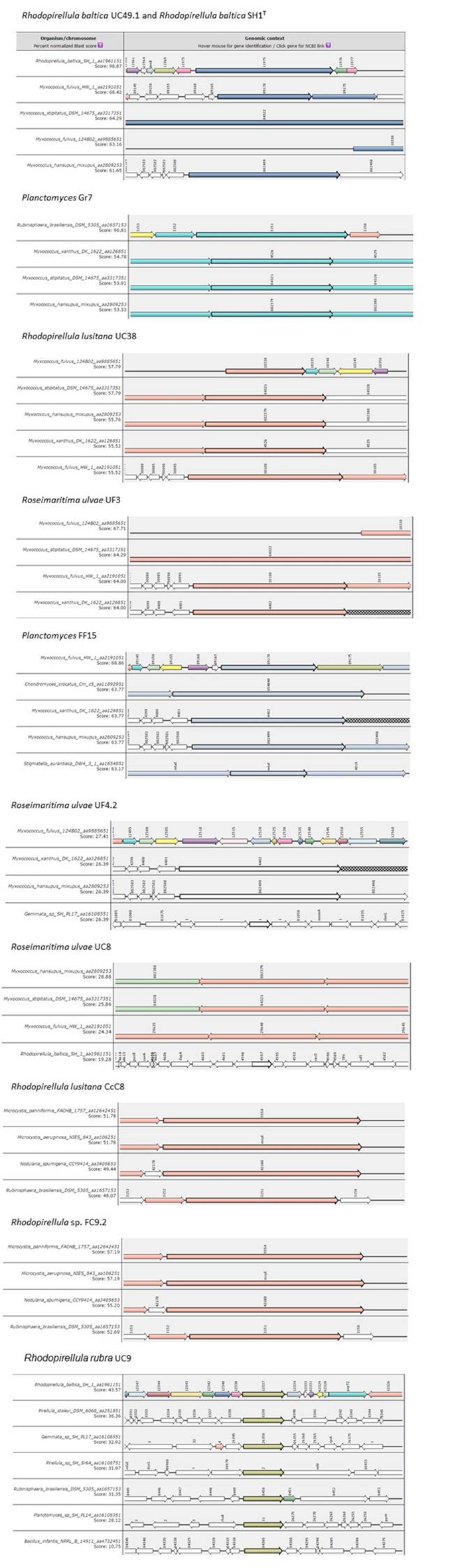

Supplement: Figure S3 — Result of the synteny analysis of Planctomycetes PKS and NRPS amplicons sequences with the closest strains as matched in NaPDoS. All the strains were also searched against Planctomycetales group for synteny. Only the results with higher similarity are shown. [file Image3.jpg]
